# Autism Diagnosis in the United Kingdom: Perspectives of Autistic Adults, Parents and Professionals

**DOI:** 10.1007/s10803-018-3639-1

**Published:** 2018-06-12

**Authors:** Laura Crane, Richard Batty, Hanna Adeyinka, Lorna Goddard, Lucy A. Henry, Elisabeth L. Hill

**Affiliations:** 1 Centre for Research in Autism and Education, UCL Institute of Education, University College London, London, WC1H 0NU UK; 2 London South Bank University, London, UK; 3 Goldsmiths, University of London, London, UK; 4 City, University of London, London, UK

**Keywords:** Autism, Diagnosis, Support, Adults, Parents, Professionals

## Abstract

Accessing an autism diagnosis is a key milestone, both for an individual and their family. Using a qualitative methodology, the current study examined the views and experiences of ten autistic adults, ten parents of children on the autism spectrum, and ten professionals involved in autism diagnosis, all based in the United Kingdom (UK). Interviewing these 30 respondents about the diagnostic process and subsequent support options, the goal was to identify aspects of the diagnostic process that are working well, and areas in which improvements are needed. Using thematic analysis, three key themes were identified: the process of understanding and accepting autism; multiple barriers to satisfaction with the diagnostic process; and inadequate post-diagnostic support provision.

## Introduction

Dissatisfaction with the process of obtaining a diagnosis of autism spectrum disorder (henceforth, autism) has been well-documented. Parental surveys have indicated lengthy delays in accessing an autism diagnosis (Crane et al. 2016; Howlin and Moore 1997; Siklos and Kerns 2007; Wiggins et al. 2006), particularly for children who are verbally and cognitively able (Crane et al. 2016; Howlin and Asgharian 1999). This can be stressful for parents (Crane et al. 2016; Siklos and Kerns 2007), and may impact on their initial reactions to the diagnosis (see Quine and Pahl 1986; Wiggins et al. 2006; Woolley et al. 1989). Parents express particular frustration with the lack of support they are offered post-diagnosis (e.g., Crane et al. 2016; Howlin and Moore 1997; Osborne and Reed 2008; Potter 2017; Siklos and Kerns 2006, 2007), which may be linked to parental expectations: while many parents assume that obtaining a formal autism diagnosis will act as a gateway to help and support, the reality is that they are often left to manage the situation alone, fighting for access to services (Carlsson et al. 2016).

For autistic adults^1^, views on the diagnostic process are more mixed. A recent survey of 128 autistic adults showed that whilst 47% were satisfied with their experiences of receiving an autism diagnosis, 40% were dissatisfied (Jones et al. 2014). This could be explained by inconsistencies in the length of time taken to obtain a diagnosis, along with varied and heterogeneous routes to diagnosis (for examples, see Hearst 2015). Mixed satisfaction with the diagnostic process may also relate to the varied demographic and cognitive characteristics of those seeking a diagnosis in adulthood (e.g., Happé et al. 2016), in addition to the presence of co-occurring mental health conditions that are common in autism (Russell et al. 2016). The situation does appear to be improving, however, with adults diagnosed more recently experiencing shorter delays (Jones et al. 2014; National Autistic Society 2012). Autistic adults do, nevertheless, express dissatisfaction with the availability and quality of post-diagnostic support, with Jones et al. (2014) reporting that just 21% of autistic adults felt satisfied with the help they were offered after receiving their diagnosis. Indeed, satisfaction with post-diagnostic support has been shown to be a key predictor of autistic adults’ satisfaction with the overall diagnostic process (Jones et al. 2014), mirroring the findings of parent surveys (e.g., Crane et al. 2016).

The views of parents and autistic adults should be considered alongside the perspectives of professionals involved in autism diagnosis, to gain a more holistic view of the diagnostic process. Indeed, to ensure the quality of the diagnostic process, expertise from several perspectives needs to be integrated: that of the individual, their family, and the professionals (De Clercq and Peeters 2007). A goal for professionals is to reduce the (often lengthy) waiting times for an autism diagnosis, whilst also providing a high-quality diagnostic service that adheres to best-practice clinical guidelines (Rutherford et al. 2016). A recent survey of 116 professionals in the UK (largely paediatricians, psychologists, and speech and language therapists) highlighted several challenges that made it difficult to provide a timely and appropriate autism diagnosis (Rogers et al. 2016). For example, professionals noted that services were not able to provide assessments as quickly as they should. They also felt that the tools used to aid diagnosis were inadequate in certain cases (e.g., when diagnosing women, or adults without learning disabilities) and that the help and support available to autistic people and their families following diagnosis (particularly over the long-term) was an area of significant concern (Rogers et al. 2016; see also Taylor et al. 2016). Disparities have also been found between the needs of parents and the practices of clinical professionals. For example, whilst 98% of parents reported that information about the meaning of the autism diagnosis and accessing relevant support services was important to them, this information was infrequently provided by professionals (Hennel et al. 2016).

The aim of the current research was to examine—in more depth than previously—autism diagnostic experiences in the United Kingdom (UK). Uniquely, we conducted semi-structured interviews with members of three key stakeholder groups: autistic adults, parents of children on the autism spectrum, and professionals involved in autism diagnosis. Interviewees were questioned about three important stages of the diagnostic pathway: (1) accessing a diagnostic service; (2) the diagnostic process; and (3) post-diagnostic support. The goal of the research was to identify aspects of the autism diagnostic process that are working well, and areas in which improvements are needed, to inform recommendations for service improvements.

## Method

### Design

This research was part of a larger project exploring the autism diagnostic process in the UK. In Phase One of the research, online surveys were developed to elicit the views and opinions of autistic adults (Jones et al. 2014), parents of children on the autism spectrum (Crane et al. 2016) and professionals involved in autism diagnosis (Rogers et al. 2016). Phase Two (presented here) comprised detailed interviews with a sub-sample of ten adults, ten parents, and ten professionals, all of whom took part in the original surveys.

Interviews were conducted over the telephone (by one of the authors, HA), before they were recorded and transcribed verbatim (via a professional transcription service). The resulting data (from adults, parents and professionals) were analysed using thematic analysis (following guidance outlined in Braun and Clarke 2006). Using this approach, data were interpreted within an essentialist framework (reporting experiences, meanings, and the reality of the interviewees), using an inductive (‘bottom-up’) approach. Specifically, data were coded without the aim of integrating themes within any pre-existing coding schemes, or preconceptions of the researchers. The only decision made in advance of the coding process was not to include data that were not directly linked to the process of autism diagnoses (e.g., reports of generic issues with healthcare services were not coded^2^). Two of the authors (LC and RB) worked independently to familiarise themselves with the data, reviewing transcripts from each of the three participant groups in turn. This involved reading and re-reading each set of transcripts several times and assigning codes to reoccurring themes, which were then organised into categories of best fit (initial themes). These preliminary themes were identified using a semantic approach; identifying themes at a ‘surface’ level, without theorizing beyond the actual content of the data. The authors then began the process of merging the themes across participant groups; identifying areas of linkage, to present an integrated overview of the autism diagnostic process. The authors met several times throughout the coding process to discuss the themes, review discrepancies and decide on the final themes and sub-themes across the stakeholder groups.

### Participants

Stratified random sampling was used to select participants to take part in the interviews. As the aim was to interview 10 participants per group (from the wider database of respondents to the three online surveys), the total number of participants from each group who were willing to be contacted for follow-up interviews was divided by 10 and every *n*th person on the list was approached via email. If they were unavailable, or if they did not respond, the next person on the list was contacted.

The parent group comprised ten mothers between the ages of 23 and 56 years (mean = 45.50, SD = 10.36). Their children (eight boys, two girls) were between the ages of 3 and 19 years (mean = 10.60, SD = 5.46). The parents initially had concerns about their children’s development when the children were between the ages of 18 months and 12 years (mean = 4.02 years, SD = 3.78). They sought help soon after these concerns emerged, when their child was, on average, 4.29 years (SD = 3.68). The mean age at diagnosis was 6.12 years (SD = 4.47, range 2–14 years), with an average delay of 1.83 years (SD = 1.78, range = 0–4.5 years) from when parents first contacted a healthcare professional to the point at which a formal autism diagnosis was received. The specific diagnoses that the children received were autism (n = 3), Asperger syndrome (n = 5) or autism spectrum disorder (n = 2). Eight of the ten children also had additional diagnoses, which included a learning disability (n = 2), a behavioural condition (n = 1), a mental health condition (n = 5), or a motor condition (n = 2). On average, the children received their diagnoses within 5 years of the research, although the range was wide (mean = 4.47 years, SD = 4.30, range = 11 months–13 years). The sample represented a number of geographical regions across the UK, with three parents from London and the South East, two from the South West and one from each of the following regions: East Midlands, North East, South West, Wales, West Midlands, and Yorkshire and the Humber. Regarding ethnicity, nine parents were from a White ethnic background, and one was Chinese.

Ten autistic adults (six women and four men) were interviewed. Of these, nine were diagnosed with Asperger syndrome and one had received a diagnosis of autism. Nine of the adults provided their ages (mean = 42.89 years, SD = 11.71, range = 29–59 years) and all were from a White ethnic background. Providing information on geographical location, five adults were from the South East, two were from the South West, and one was from each of the following: East Midlands, West Midlands and the North West. Questioning participants about their diagnosis, all but one were diagnosed in adulthood (mean age = 38.90, SD = 13.79, range = 10–57 years). The average age at which they (or someone else) first suspected they may be not be neurotypical was 33.10 years (SD = 17.82, range = 2–56 years), and the average delay from first contacting a healthcare professional to receiving a formal diagnosis was 1.71 years (SD = 1.89, range = 1–6 years). Six of the ten adults had additional diagnoses: attention deficit hyperactivity disorder (ADHD) (n = 1); anxiety (n = 5); depression (n = 5); dyslexia (n = 1); dyspraxia (n = 3); obsessive compulsive disorder (OCD) (n = 3); and personality disorder (n = 1). Of the nine adults who provided the length of time since their autism diagnosis (in the original survey), seven were diagnosed within the last 5 years (mean = 6.56, SD = 10.16). Gathering further information about the characteristics of the sample, six of the ten adults lived at home with their partner and/or children, and four lived at home with their parents and/or siblings. Questioning interviewees about their highest educational qualifications, three adults were educated to postgraduate level, one to undergraduate/bachelor degree level, two to A Level standard (16–18 years), and three to GCSE/O Level standard (14–16 years). One participant was awarded an advanced diploma (a professional qualification, just below the level of an undergraduate/bachelor degree). Regarding current employment status: four adults were not currently employed, nor were they looking for work; three adults were undertaking voluntary work; two adults were in paid full-time work; and one adult was in full-time education.

Ten professionals (eight women and two men) who were involved in autism diagnosis took part in the interviews. This sample comprised three clinical psychologists, two paediatricians, one educational psychologist, one psychiatrist, one speech and language therapist, one specialist early years practitioner, and two educators. The professionals were relatively experienced in their professional roles: two had less than 5 years’ experience, three had 5–10 years’ experience, four had 10–15 years’ experience and one had over 20 years’ experience. Seven professionals worked for the UK’s National Health Service, two worked in the education sector, and one worked for a local authority. Asked about the age groups that they worked with, eight specialised in child diagnosis, whilst two specialised in adult diagnosis. The sample was geographically diverse, with three professionals from Yorkshire and the Humber, two from London and the South East, two from Scotland, and one from each of the following: East Midlands, North West England and Northern Ireland. Providing information on ethnicity, nine were from a White ethnic background, with one from a Black ethnic background.

### Materials and Procedure

Ethical approval for the study was granted by the Research Ethics Committee within the Department of Psychology at Goldsmiths, University of London. All participants gave informed consent prior to participation in the semi-structured interviews, which were conducted by one of the authors (HA). The interviewer explained to the participant that they did not have access to the questionnaire data that they originally provided, ensuring the interviewees were aware of the need to describe their experiences fully and not to rely on information previously provided.

An interview protocol was used to guide the interviews. All protocols began with a rapport building phase, in which the interviewer provided the interviewee with details about the nature and purpose of the interview. For adult and parent participants, the protocol covered the following topics: initial concerns and the process of starting to seek an autism diagnosis; the diagnostic process and their initial expectations; the consultation in which the autism diagnosis was confirmed; the information provided at the diagnostic consultation; emotions and feelings about the process of diagnosis, as well as associated coping strategies; and, finally, met and unmet post-diagnostic support needs. Following this, the interviewer probed for both positive and negative aspects of the diagnostic process, and asked about any aspects of the process that could be improved in future.

For professionals, the interview protocol began with a discussion of their role and the contact they have with people on the autism spectrum and their families. The protocol was then divided into three key sections (as per Rogers et al. 2016). Section 1 focused on recognition and referral (i.e., accessing a diagnostic assessment), in which interviewees were asked for their thoughts on the accessibility of services, and to comment on key issues raised in the original survey (including any impact of increasing caseloads, resources, timeliness of assessments, and barriers to services). Section 2 covered the diagnostic process and decision making, in which interviewees were asked to comment on diagnostic tools, the degree to which they relied on professional judgement, and how they disclosed a diagnosis. Section 3 focused on post-diagnostic support, including availability and satisfaction with the provision offered to the people and/or their families that they worked with. In all three sections, there was a focus on highlighting both positive and negative aspects of the diagnostic process, in addition to areas that needed improvement (and potential solutions).

All topics of discussion began with open questions, to allow the respondent to provide their views and perspectives without any undue influence from the interviewer. All interviews were conducted on the telephone and the mean length of the interviews was: 51.54 min for the autistic adults (SD = 19.22, range = 22–96 min), 42.10 min for the parents (SD = 13.24, range = 30–71 min) and 51.22 for the professionals (SD = 14.03, range = 32–75). Some interviewees (two adults, two parents and two professionals) also took part in short (20 min) follow-up interviews, following the analysis of their initial interview, to explore in more detail some of the points they originally raised. Data from these short follow-up interviews were then integrated with data from the main interview, for subsequent analyses.

## Results

**Fig. 1:**
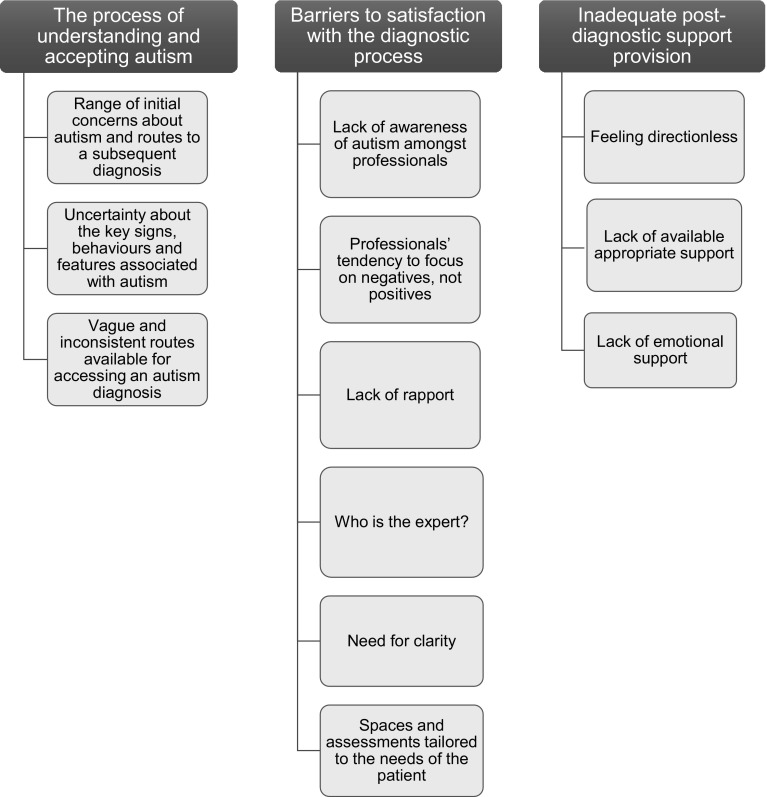
Themes and subthemes discussed by autistic adults, parents of children on the autism spectrum and professionals involved in autism diagnosis

Three main themes were identified from the interview data: (1) the process of understanding and accepting autism; (2) barriers to satisfaction with the diagnostic process; and (3) inadequate post-diagnostic support provision (see Fig. 1).

### Theme 1: The Process of Understanding and Accepting Autism

Parents reported a **range of initial concerns and routes to a subsequent diagnosis**; highlighting problems with speech (e.g., delays in reaching typical milestones) and behaviour (e.g., tantrums), as well as general developmental delays (e.g., late toilet training). Parents who had older typically developing children also noted stark differences between the development of their autistic and non-autistic children. However, parents were often in denial at this early stage: “I kept telling myself he’s delayed but he’ll come along fine and he just needs a bit more time…which, in hindsight, was not a good choice, not a good start.” (parent). Parents acknowledged the need for perspective: “I know it’s not the hugest—it’s not like your child has leukaemia or your child has a congenital heart defect or something.” (parent); yet still felt that this was a fairly “profound” milestone for both their child and the family: “I don’t think that was taken into account, how different you feel when someone does confirm it.” (parent).

Adults reported accessing a diagnosis via a range of routes (e.g., following a crisis; following many years of experiencing difficulties without understanding the reasons behind these; following their children receiving an autism diagnosis). For some, the diagnosis came as a complete surprise: “I was just expecting support with my mental health. I wasn’t expecting to be diagnosed with autism.” (adult); for others, the process was entered with the specific intention of having their suspicions of autism formally confirmed by a clinical professional: “I knew I’d got Asperger’s. I didn’t need, really, somebody to tell me. I was just gaining the rubber stamp.” (adult). As also noted by the parent interviewees, adults viewed the confirmation of the suspected diagnosis as significant: “It didn’t change what I already knew but then if you need to tell your diagnosis for some reason, whether that’s for employment or for disability allowance or for access to support or for taking part in research, you’ve got it there and it’s proof and you can do the things you need to do with it…it’s validation for yourself as well.” (adult).

Many interviewees (particularly parents, but also some adults) reported **uncertainty about the key signs, behaviours and features associated with autism**. Parents reported limited knowledge of autism, and felt naïve about what to expect during different stages of the diagnostic process. Even autistic adults who had good knowledge of autism and atypical development often failed to notice their own autistic features or behaviours: “I work with people with autism…my oldest child has got Asperger’s…but I didn’t recognise it in myself.” (adult).

For parents, the concept of their child being ‘different’ could, initially, be difficult to accept: “Parents want a normal child. Honestly, they don’t want a child who is different.” (parent). The concept of autism as a life-long condition also caused concern for parents: “I asked a few basic questions and [the clinician] said it will be something he has for life. I was very shocked because I thought that if he had some mental illness it would go away and this seemed to be a disability that he would have to live with rather than a shorter-term thing.” (parent). These concerns did not, however, negatively impact on parents’ views of their children: “She’s still my daughter first and foremost, the fact that she has autism is secondary to that.” (parent).

Some interviewees noted the stark difference between the clear and supportive pathways they previously experienced for medical diagnoses, relative to the **vague and inconsistent routes available for accessing an autism diagnosis**. This was noted by parents: “My mother had cancer and when she got her diagnosis she was told, right, this is the clinic you attend, this is the doctor you have, this is the nurse’s helpline you have; [she] was told a course of events… and that’s not what happened to us at all.” (parent); as well as adults: “[on getting diagnosed with a serious medical condition] there was a trained nurse who sat down with me for an hour and talked through it, the consultants were really good about it and spoke to me and the contrast was quite amazing compared to what happened around my autism diagnosis… It was cold, it was calculating and there was nothing else there, as if they didn’t see it as any sort of potential issue in my life really, it was just yes or no.” (adult).

### Theme 2: Barriers to Satisfaction with the Diagnostic Process

Several barriers to satisfaction with the diagnostic process were noted. One major barrier was a perceived **lack of autism awareness amongst professionals**, particularly in relation to General Practitioners (GPs; family doctors) and teachers. GPs were often reported to be the first port of call for parents and adults seeking an autism diagnosis, yet: “it seems that GPs aren’t trained in awareness of Asperger’s syndrome.” (adult). This created anxiety at an already stressful time: “I’d never brought anything up like this with the GP before…I felt completely at the mercy of whether they believed me or not…whether they agreed with my assessment of myself…I was really very worried.” (adult). Interviewees did, however, acknowledge examples of good practice: “…the second GP I saw was much more helpful and understanding and so when I go back to my GP surgery, if it’s anything to do with Asperger’s, I insist on seeing him.” (adult).

Parents noted similar issues amongst school and nursery staff. At minimum, it was felt that school staff should be aware of who to refer parents to, should concerns be raised about their children’s development, yet parents often had to instigate referrals themselves and navigate the process alone. As one parent explained: “the teachers [refused] to have him assessed…I decided to put in an application myself for parental request for assessment. So I completed all the forms and I put about his emotional difficulties and his processing things, also sensory difficulties and when I look back now, I’m horrified that the local authority didn’t pursue it because it absolutely shouted autism”.

Despite calls for increased awareness and training regarding autism amongst frontline health and education professionals (e.g., GPs, teachers), interviewees highlighted the need for balance, and for this to be managed sensitively: “You sometimes get teachers who, because someone puts their hands over their ears when they hear a loud noise, for instance, will have heard that as one of the factors that may be associated with autism but then will start telling parents ‘have you ever thought your child might have autism?’ and it’s not that, there’s a variety of reasons for why a child might their hands over their ears when they hear a loud noise and it’s not always autism.” (professional).

Another factor contributing to the perceived barriers to satisfaction with the diagnostic process related to **professionals’ tendency to focus on negatives, not positives**; as one adult remarked: “Nobody wants their negative side highlighted all the time”. Parents emphasised several benefits of professionals focusing on the positive aspects of their case including: strengthening self-esteem and increasing positive thinking about the assessment process; making parents feel that their parenting has been worthwhile; reducing feelings of failure; undermining negative beliefs that parents hold (e.g., thinking they may be somehow to blame); and increasing trust between parents and professionals. Professionals acknowledged the need to frame the diagnosis in a positive way: “…you reassure them this doesn’t change the child you have, the child is still the same child, in fact, your child’s difficulties have always been there. All we have done is to put a name to the difficulties…and you try and let them know their strengths, that there is something good also about their child that can be built on.” (professional). Nevertheless, parent and adult interviewees reported that the emphasis was very much on what the autistic individual could *not* do: “She may have [mentioned some positive aspects of autism]—it may just be that I remember the more negative stuff…if the way I’m remembering it is accurate, she could have been a bit more encouraging.” (adult). Interviewees called for any recognition of difficulties to be accompanied by practical strategies for support and assistance: “They listed all the findings that he was struggling with but they never suggested or they never carried out any ways of trying to address those.” (parent).

Issues such as these contributed to a general **lack of rapport** between adults/parents and professionals: “The most important thing for any family going through it (an assessment) is how you are made to feel in the beginning, the rapport you have.” (parent). Many professionals agreed that training in rapport building and empathy was “really important” and that “establishing a bond should be the clinician’s first objective because it directly impacts on the amount and quality of information they give us.” (professional). Some did, however, stress the need for caution in the rapport building phase: “When you show too much understanding or empathy, people want to spend a great deal of time going into their stories, although I would happily listen, I just don’t think I have the time.” (professional).

Contributing to the patient-professional tension was debate concerning **who is the expert?** Many adults and parents entered the diagnostic process ‘knowing’ that they were autistic, or that their child was on the autism spectrum. Indeed, one adult persisted with multiple assessments until they finally received an autism diagnosis: “The [professionals] said I didn’t have [autism] and I knew that they’d got it wrong…although they were meant to be like the experts, I really didn’t feel like they were…” (adult). Many professionals cited concerns over such behaviours, highlighting how this could lead to the patients’ needs not being properly addressed: “When parents come to the assessment with this attitude, and it is very common, it is a big problem. I think it makes them resistant to other possibilities and diagnoses, so resistant and they shut down. The real problem is that the child’s real needs may not be addressed properly.” (professional). Irrespective of the outcome of the diagnostic assessment, it is important to ensure that professionals provide advice and support to their patients and their families: “…it is about telling parents that we will still offer the support that is needed, but not make false diagnosis just because you need [a label]—the parents need support.” (professional).

Further, while it is crucial to appreciate the expertise professionals offer, it is essential to also appreciate the expertise that autistic adults and parents bring: “The main thing is making parents feel they are being listened to and taken seriously…I was made to feel that I was the expert on my child and that’s important because if you’re belittled and made to feel that you don’t know what you’re talking about, then the relationship breaks down.” (parent). Working in partnership is key: “I was told many times, leave it to the professionals, which is an absolutely abysmal statement on two grounds: one is it’s really a very crude insult and two, they’re only professionals if they do their job really well and in most cases, in our particular case, they didn’t…the best way is to work in partnership…you need to be on the same side and you need to all be working in the same direction.” (parent).

For parents and adults, there was a **need for clarity** during the diagnostic process and a relative lack of this was a key barrier to satisfaction. Some interviewees complained about the use of clinical language: “[you need] some kind of glossary of abbreviations…I just felt completely clueless.” (parent). Professionals reported that they were aware of this and that they try to address the issue: “We make the effort…we speak in very, very layman’s terms, we avoid the medical jargon and make it very simple for them to understand.” (professional); and, when this happened, it was responded to favourably: “He was using quite a lot of long words but he was aware of that and he would stop and explain what they meant. He wrote things down as well, which was useful so we could take that away.” (parent).

For parents and adults, more transparency was needed concerning the whole process. This included the role of the professionals involved: “Only now, looking back, can I see the big picture—how it all fits together, how all the people involved in the system work together. It’s a big system…at the time, I didn’t know who was who, what they did or anything.” (adult); as well as post-diagnostic support available: “If I hear exactly what they can offer me and can’t offer me, I would feel happier. I’d know, after this meeting, I will be expected to do my own research, and speak to other families, and find my own support groups…” (parent).

A further barrier to satisfaction with the diagnostic process regarded the **spaces in which the assessments were carried out, and the activities the patients were required to do during the assessment**. In addition to the sensory demands (“the light, my goodness, it was too much”; adult), the environment was not always considered appropriate for the patient: “The waiting area was more for adults than for kids.” (parent). Likewise, some of the materials used in the assessment were not pitched at the right level and this was deemed patronising: “I was aware that those questionnaires were part of the process and I’ve spoken to a lot of people on forums who feel exactly the same way, that they’re kinda aimed at children…I didn’t particularly feel like a 40-year-old being questioned.” (adult).

### Theme 3: Inadequate Provision for Post-diagnostic Support

Adults and parents reported **feeling directionless** after they had received the formal autism diagnosis: “[we needed] more information of where you can get help rather than just sort of…be dumped after the diagnosis.” (parent). Parents, in particular, complained about the lack of a follow-up appointment with the diagnosing professional or a member of their team: “Getting the diagnosis is only the start of the journey and as far as the paediatrician was concerned, that was the end of the journey.” (parent); and felt that information needed to be more readily offered to autistic people and their families: “[you need someone] to support you and direct you…this is where the system fails.” (parent). This lack of advice and guidance was particularly difficult given that parents and adults were often struggling to make sense of the diagnosis and what it meant for them and their family. As one adult explained: “I don’t think diagnosis in itself often helps you…[Where I work] we profile people after they’ve been diagnosed so that they can understand how they are, and who they are, and look at where their weaknesses are and when their strengths are”.

Both parents and adults expressed dismay at the general **lack of appropriate support** following the diagnosis. When services were made available to support autistic people and their families, these tend not to be offered until crisis point was reached: “Everything’s been a fight. Everything’s had to be, you turn up on the professional’s doorstep and go, I’m drowning—what can you do to help? And they go, oh, don’t you know about x, y and z? No! There’s no continuous care.” (parent). Even more worryingly, when support was provided (and was felt to be useful), financial constraints sometimes meant that services were withdrawn: “We were going to this early autism school project [and] it just suddenly stopped and I felt like I’d lost a lifeline there…it was like, oh, where do I go now?” (parent).

A further difficultly was finding appropriate post-diagnostic support specific to adults: “I found an [autism] group meeting, and I walked in and it was little kids with their parents all having cups of coffee in a play centre…I’m 46, I don’t want to go on a climbing frame with 4-year-olds.” (adult). This was especially true for adults without intellectual disabilities: “…it all seems to be geared to people with much more severe problems. There’s just not a lot of help unless you’re very visibly autistic.” (adult). The spectrum nature of the condition was also highlighted as a key barrier to accessing appropriate support: “[The support group] was too stressful ‘cause it’s too much of a wide spectrum of people with autism. There was people with so many different types of needs...problems and different ages...very stressful.” (adult).

Adult interviewees were clear about the kind of support that they wanted post-diagnosis, particularly practical advice and guidance: “More help with benefits, education, employment, housing, healthcare, you know, every aspect of my life.” (adult). When adults were offered appropriate post-diagnostic support, this was viewed very positively, yet they longed for more organised and overarching support: “I wish that there was a solid, government-backed organisation that could help rather than these sort of variegated charities that are all struggling financially.” (adult).

Professionals were aware of the issues surrounding a lack of appropriate post-diagnostic support. Whilst they wanted to offer such support, they were often unable to: “We’re put under increasing pressure not to offer significant post-diagnostic support because of the multiple pressures on our service.” (professional). Managing expectations seemed to be key here: “There is this perception that there’s going to be a huge amount intervention, a really quite substantial level of support when, in fact, a lot of the time it’s going to be up to them…you’d like to be offering more but the reality is that it’s more about helping them help themselves.” (professional).

The combination of a general lack of support and poor signposting to relevant services was often exacerbated by a **lack of family support**. Parents noted that some family members did not agree with the child’s autism diagnosis, which caused tensions: “My husband’s parents were particularly of the breed of, ‘oh I wouldn’t let him get away with that’, ‘oh, look at him, he’s kicking off in a supermarket for no reason, that’s terrible, you never did that’…they were just expecting things of him that he just couldn’t achieve.” (parent). As one professional sympathised: “If somebody’s in a wheelchair or having difficulty walking, you can see that from the outside, whereas autism is invisible from the outside and I’ve people who struggle with that whole concept…it is actually a disability, it is actually a real issue.” (professional).

Whilst a lack of family support was, understandably, upsetting and difficult for parents, it seemed to have a far greater impact on autistic adults. Not all adult interviewees had the support of family members during the assessment process; in fact, many experienced tensions with some family members who refused to accept the autism diagnosis: “My mother and father won’t accept [my diagnosis]…[they accepted my son’s diagnosis] after a long time, but my mum will still produce newspaper clippings of what an autistic child is and she’ll say, that’s not what [child] is, he’s not autistic, he can’t be.” (adult). Some adults were grateful for support from their families, but noted that tension had arisen prior to the diagnosis due to a lack of understanding: “I have a very understanding partner and I have a very understanding family but we’ve had conflicts in the past because they didn’t fully understand me, I didn’t fully understand myself.” (adult). Conversely, others noted that the diagnostic process itself was made easier by having support at home: “the key thing was that I had a family who were supporting me.” (adult).

Support from other people who had been through the same experience was also reported as helpful: “The best support and advice I have got is from meeting other parents, not the medical profession at all.” (parent). However, interviewees acknowledged that there can be issues with parents supporting other parents: “If that’s what the state relies on for support, then that’s just hopeless because it’s just loads of people helping each other who are on the edge, on the verge of a nervous breakdown.” (parent).

One further barrier to effective relationships was the perceived **lack of emotional support**. Parents reported many different (often negative) emotions—anger, blame, shock, guilt, sadness, worry, stress, relief—and felt that these feelings were, for the most part, “ignored” by professionals. Navigating through the diagnostic process was felt to exacerbate difficulties parents were experiencing: “I was literally on my knees anyway…it’s so tiring having boys with Aspergers.” (parent); and was seen as very isolating: “I felt quite on my own. No-one in my family really understood, I didn’t have any friends that had had similar experiences.” (parent). Yet managing parental stress was found to be beneficial for the child and was, potentially, something that parents felt professionals should have more responsibility for: “If I’m coping, if I’m managing my stress, then surely my daughter will be in a better place too? Do professionals not have a responsibility there?” (parent). Some professionals noted that their services offered “whole family support needs” in addition to aspects more tailored to the child, such as “self-care/adaptive needs, behavioural and emotional needs, learning needs, and communication needs” (professional). However, this did not appear to be something consistently available across all services.

A lack of emotional support was an issue for adults, given that many found their involvement in the assessment process both emotional and challenging. For example, many interviewees, although they did expect professionals to ask them about their past and their childhood experiences, found this aspect of the process extremely difficult, as they did not expect to be probed so extensively on these points: “I found it quite traumatic…they [professionals] are dredging up old stuff, things from the past…then you’re just left to work all that out for yourself.” (adult). On some occasions, this raised issues for the broader family: “It took me and my mum months to work through all of that afterwards…I think my mum used to feel very guilty about me having a hard time when I was a kid, not fitting in, and then this man just goes and brings all those feelings back for her…it was a nightmare.” (adult). After raising these tensions, the adults and their families found themselves with no support to manage these issues: “It’s like the flood gates opening and you’re left to just drift.” (adult).

Other adults found the history gathering phase of the assessment helpful, with family members seeming to benefit from revisiting the past: “It was a mirror showing my parents how it was, their part in my upbringing, and I thought that was very good because I wasn’t always happy with the way my parents raised me. The assessment allowed me to be able to express this afterwards to my parents, and my parents said they had no idea…It was quite therapeutic and we started talking more openly after the assessment.” (adult). It also raised awareness of autism within the broader family: “The diagnosis process brought into light that my father and grandfather had quite similar developmental histories to me…It was also quite a bonding experience for the male line in my family…later on, my brothers went through a similar process.” (adult).

## Discussion

Interviewing autistic adults, parents of children on the autism spectrum and professionals involved in autism diagnosis—and integrating their knowledge and experience—offered important insights into the autism diagnostic process in the UK. Specifically, three key themes emerged from the interview data: (1) the process of understanding and accepting autism; (2) multiple barriers to satisfaction with the diagnostic process; and (3) inadequate provision for post-diagnostic support.

Many interviewees reported initial uncertainty about both autism and the diagnostic process. Awareness of autism has been growing, with most people in the general population being aware of autism or knowing someone on the autism spectrum (Dillenburger et al. 2013). Yet despite the public having relatively accurate knowledge of the profile of strengths and weaknesses associated with autism (Dillenburger et al. 2013) it may not be easy to identify signs of autism in very young children (especially for first-time parents) or in those who may not ‘fit’ the standard descriptions of autism, such as adults who do not have intellectual disabilities, or women and girls (see, for example, Gould and Ashton-Smith 2011). It is, therefore, important to focus research efforts on exploring early knowledge-seeking behaviours amongst parents and adults, particularly since caregivers often rely on the media, conferences and other parents to find out more about autism (Rhoades et al. 2007); accessing information that may not necessarily be accurate. One must, however, strike a careful balance between supporting parents and/or adults to accurately identify autistic-like features as early as possible, and not causing unnecessary concern amongst those who do not meet criteria for autism but may show some isolated autistic-like features.

When adults and parents did identify initial signs of autism in themselves or their children, several barriers to accessing an autism diagnosis were noted, and these emerged from the very early stages of the process. In particular, there was consensus—across all three participant groups—that there needed to be far greater awareness and training about autism for frontline healthcare and educational professionals, particularly GPs and teachers. Unigwe et al. (2017) recently surveyed GPs in the UK about their experiences of working with their autistic patients. Whilst the GPs in their sample had good knowledge of autism, they lacked confidence in their ability to identify and support their autistic patients. Further, the GPs highlighted several barriers (e.g., a lack of support for autistic adults and families, lengthy delays between referral and diagnosis) that were highlighted in the current research and in previous studies on autism diagnosis (e.g., Crane et al. 2016; Jones et al. 2014). Encouragingly, the Royal College of GPs—the UK’s professional body for GPs—has recently developed a range of resources to support GPs in the identification and management of their autistic patients. This is essential, as the complex healthcare needs of autistic people require a “skilled general practice workforce” (Foley et al. 2017, p. 1). This complements similar initiatives for supporting teachers—another key frontline professional group—in working with autistic students (which will be a compulsory aspect of initial teacher training in England from 2018). Whilst it will take time for initiatives such as these to impact positively on autistic people and their families—and there is a need for continual monitoring and evaluation of the efficacy of such training and its impact on practice—they are certainly positive steps in the right direction in the UK.

Professionals do, however, often cite systemic factors (e.g., a lack of funding, increasing caseloads and limited resources) as barriers to providing effective services to their autistic patients and their family members (e.g., Rogers et al. 2016; Unigwe et al. 2017; Ward et al. 2016). Indeed, many professionals in the current study wanted to provide greater care and support to their patients (particularly post-diagnosis) but were unable to for such reasons. It is encouraging that many of the issues noted in the current interviews—such as a focus on positive aspects of autism, efforts to improve rapport between patients and professionals, and mutual respect (in relation to the expertise that adults, parents and professionals bring)—do not necessarily require financial commitments or radical overhauls of existing service provision (as per Nicolaidis et al. 2014). That does not, however, mean that they are easy to implement. Professionals face many tensions in maintaining a position of honesty about the challenges that autism can bring, without being too pessimistic. At early stages of development, in particular, it is difficult (if not impossible) to predict a child’s outcomes in later life and a balance needs to be struck between providing optimism to families and raising expectations too high. For professionals, taking the time to explain the way the autism diagnostic process is approached (i.e., that diagnostic manuals focus on highlighting the challenges that autistic children, young people and adults face) could provide important context to why difficulties are the main focus of the assessment.

A further key tension, which was frequently commented on during interviews with all stakeholders, regarded debate over who the ‘expert’ was in the diagnostic situation. De Clercq and Peeters (2007) highlight the importance of bringing together expertise from patients, families and professionals. However, the results of the current study suggest that this balance is not always an easy one to strike. Professionals, in particular, highlighted many issues with adults and parents entering the diagnostic process ‘knowing’ that they, or their child, were on the autism spectrum; and professionals cited concerns that this resulted in the individual or their family being reluctant to explore other diagnostic possibilities. Reasons for some adults and parents so adamantly seeking a diagnosis were often unclear (although themes of self-identity and access to support were touched upon). Further research is necessary to examine the perspectives of individuals and families who do not leave the diagnostic process with confirmation of an autism diagnosis, to identify (more systematically) how to address this tension.

There was, however, consensus across all stakeholder groups that the process of autism diagnosis needed to be more respectful, accessible and patient-centred (see also Nicolaides et al. 2015). Interviewees noted two specific areas where this needed to be addressed. First, care needed to be taken to ensure that assessment centres, assessment rooms, and waiting areas are made more appropriate and accessible for autistic people, to contribute towards easing their fears and anxieties about the diagnostic process (see also Nicolaides et al. 2014). Second, the assessments themselves, as well as the post-diagnostic support options given to patients, needed to be pitched at the right level. This will not be an easy task; particularly in relation to autism assessments in which ‘gold-standard’ diagnostic assessments are administered according to strict protocols (e.g., Lord et al. 2012; Rutter et al. 2003/2008). However, it is important to listen to, and respect, the views of autistic people and their families and explore ways in which positive accommodations can be made as much as possible (see Nicolaides et al. 2014, 2015).

The final theme that emerged from the interviews was inadequate post-diagnostic support provision. In line with the results of large-scale surveys (Crane et al. 2016; Jones et al. 2014; Rogers et al. 2016), a general lack of support was noted for both autistic individuals and their families following confirmation of an autism diagnosis. Professionals acknowledged the general lack of post-diagnostic support available to their patients and their families, and appreciated that they needed to become more aware of the services and support available in their local area. This is a challenging task given that such services are often provided by charitable organisations, are dependent upon the renewal of fixed-term funding, and may be transient in nature. Consideration does, however, need to be given to more subtle forms of support. Adults and parents highlighted the wider effects of the diagnostic process on the family, and it is unclear whether professionals were fully aware of the impact their questioning had on some individuals and families. Professionals must be conscious of the potential impact of the diagnostic process on an individual and their family, and consider ways to manage the consequences of an emotional and often difficult diagnostic journey.

The key strength of this study was the in-depth exploration of the autism diagnostic process from the perspectives of three key stakeholder groups (autistic adults, parents of children on the autism spectrum, and professionals). However, this study is not without its limitations. First, the data are from a small sub-group of participants, all of whom needed to agree to take part in a telephone interview. The use of telephone interviews may have been a particular barrier for the autistic adults and limits the generalisability of the findings. Yet the fact that the findings so closely echo those of other research studies (e.g., Foley et al. 2017; Nicolaides et al. 2015) provides confidence in the results. Second, the characteristics of the sample warrant mention. Despite the samples being geographically diverse, there was a lack of ethnic diversity; and it has been well documented that those from ethnic minority communities may be particularly disadvantaged when it comes to autism diagnosis (Begeer et al. 2009; Mandell et al. 2002). Additionally, more women took part in the interviews than men. Whilst this was found across all of the participant groups and is likely to reflect a more general trend of women being more likely to volunteer for research studies than men (Rosenthal and Rosnow 1975), it is particularly notable in the adult group considering the higher ratio of males to females diagnosed with autism (Loomes et al. 2017). Future research is needed to more fully explore the unique factors affecting the autism diagnostic process across genders (e.g., Begeer et al. 2013) and in different minority groups (e.g., Fox et al. 2017) to identify issues specific to these groups. Finally, whilst most parents and adults encountered the diagnostic process fairly recently (receiving a diagnosis for themselves or their child within 5 years of the research), important guidelines regarding autism diagnosis in children and adults (e.g., National Institute of Clinical Excellence 2011, 2012) have been published subsequently. It is, therefore, important to replicate this research in future, to ascertain whether the implementation of such recommendations have led to positive changes in clinical practice (cf. McKenzie et al. 2016).

In conclusion, the use of a qualitative methodology with three key stakeholder groups—autistic adults, parents of children on the autism spectrum, and professionals involved in autism diagnosis—has provided a unique and detailed exploration of the autistic diagnostic process in the UK (highlighting both strengths and weaknesses). The process of accessing an autism diagnosis can be a difficult time for autistic individuals and their families, and it is essential to identify ways to better support them during all stages of the process—from when the early signs of autism are first noted, through the diagnostic process itself, to the provision of post-diagnostic support. Professionals play an important role here, and it is important that they feel confident and supported in providing healthcare that is respectful, accessible and, importantly, person-centred to their autistic patients and their families (Nicolaidis et al. 2015; Unigwe et al. 2017).

## Footnotes

1 The use of identify-first language (e.g., ‘autistic adult’), opposed to first-person language (i.e., ‘adult with autism’), is preferred by many autistic people and their allies. To respect this, we largely use identity-first language in this manuscript (see Kenny et al. 2016; and also Gernsbacher 2017).

2 Note that, as a result of this decision, quotes from professionals are under-represented in the “Results” section of the paper. This was because professionals (as opposed to parents and/or adults) tended to highlight factors such as generic funding/resource constraints in the healthcare system when discussing barriers and facilitators to autism diagnoses.
